# Chemotherapy-induced peripheral neuropathy (CIPN): current therapies and topical treatment option with high-concentration capsaicin

**DOI:** 10.1007/s00520-021-06042-x

**Published:** 2021-02-23

**Authors:** Christian Maihöfner, Ingo Diel, Hans Tesch, Tamara Quandel, Ralf Baron

**Affiliations:** 1 Department of Neurology, Fürth General Hospital, Jakob-Henle-Straße 1, 90766 Fürth, Germany; 2 Praxisklinik Am Rosengarten, Mannheim, Germany; 3 Department of Oncology, Bethanien Hospital, Frankfurt am Main, Germany; 4 Grünenthal GmbH, 52078 Aachen, Germany; 5 Division of Neurological Pain Research and Therapy, Department of Neurology, University Hospital of Schleswig-Holstein, Campus Kiel, Kiel, Germany

**Keywords:** Chemotherapy-induced peripheral neuropathy, Cancer, Neuropathic Pain, Capsaicin

## Abstract

Cancer diagnosis and treatment are drastic events for patients and their families. Besides psychological aspects of the disease, patients are often affected by severe side effects related to the cancer itself or as a result of therapeutic interventions. Particularly, chemotherapy-induced peripheral neuropathy (CIPN) is the most prevalent neurological complication of oral or intravenous chemotherapy. The disorder may require dose reduction of chemotherapy and is accompanied by multiple symptoms with long-term functional impairment affecting quality of life (QoL), e.g., sensory and functional deteriorations as well as severe pain. Although CIPN may reverse or improve after termination of the causative chemotherapy, approximately 30–40% of patients are faced with chronicity of the symptoms. Due to the advantages in cancer diagnosis and treatments, survival rates of cancer patients rise and CIPN may occur even more frequently in the future. In this review, we summarize current recommendations of leading national and international societies regarding prevention and treatment options in CIPN. A special focus will be placed on current evidence for topical treatment of CIPN with high-dose capsaicin. Finally, an algorithm for CIPN treatment in clinical practice is provided, including both pharmacologic and non-pharmacologic modalities based on the clinical presentation.

**Supplementary Information:**

The online version contains supplementary material available at 10.1007/s00520-021-06042-x.

## Introduction/background

According to the International Association of the Study of Pain (IASP) neuropathic pain is defined as pain caused by a lesion or disease of the somatosensory (peripheral and/or central) nervous system and comprises a loss of sensory function or sense as well as an increased pain sensitivity or spontaneous pain [1, 2]. Cancer-related neuropathic pain may result from direct infiltration of the primary tumor or metastases or from cancer treatments, including surgery, radiotherapy, and chemotherapy [3]. Particularly, chemotherapy-induced peripheral neuropathy (CIPN) represents a common and disabling side effect of tumor treatment with neurotoxic antitumor agents causing damage to peripheral nerves (sensory, motor, and autonomic). Although neurotoxicity levels of these substances vary, some are known to be associated with an increased risk of CIPN, e.g., platinum derivatives, taxanes, vinca alkaloids, eribulin, bortezomib, and thalidomide [4–6].

Symptoms of CIPN usually decline after termination of the neurotoxic therapy. However, depending on the administered substance (e.g., platinum compounds), further progression or new development of symptoms for several months post-therapy is possible (coasting phenomenon), and symptoms can persist for many years or even lifelong [7]. Since certain chemotherapeutic agents (e.g., cisplatin) remain in the body for a long time [8], CIPN symptoms can also develop and manifest years after completion of chemotherapy. Thus, even chemotherapies from long ago should be considered at diagnosis of emerging neuropathies, especially in conjunction with further predispositions (e.g., genetic, environmental, and life-style factors such as alcohol abuse). Some neurotoxic chemotherapeutic agents cause both acute and chronic forms of neuropathy. As an example, the frequent acute form of oxaliplatin-induced peripheral neuropathy (mainly cold-induced distal paresthesia, dysesthesia, or pain) normally reverses within a week. The chronic cumulative form persists between and after treatment. While in most patients, the severe chronic form resolves within approximately 13 weeks after treatment, a considerable number of patients still experience chronic neuropathic symptoms after more than a year [9]. The severity of chronic neuropathy seems to correlate with that of acute symptoms, and an early development of cold hyperalgesia has been suggested as predictor of severe chronic oxaliplatin-induced CIPN [10].

Approximately 50–90% of patients under chemotherapy are affected by CIPN and bear a high risk of chronicity (approx. 30–40%) [11–13]. A recent meta-analysis published by Rivera et al. revealed that CIPN-associated neuropathic symptoms persist in 11 to > 80% of early-stage breast cancer patients for 1–3 years following therapy. Particularly in light of the excellent survival prognosis of this patient cohort, it is essential to consider CIPN-related impairments in quality of life (QoL) [14].

Sensory nerve fibers are generally more vulnerable to toxic effects of anticancer drugs and patients affected by CIPN usually experience greater sensory than motor or autonomic symptoms. CIPN-specific symptoms commonly manifest in feet and hands in typical sock and/or glove-like pattern. They may cause adjustments of therapy as well as subsequent reduction in treatment efficacy [11].

**Fig. 1:**
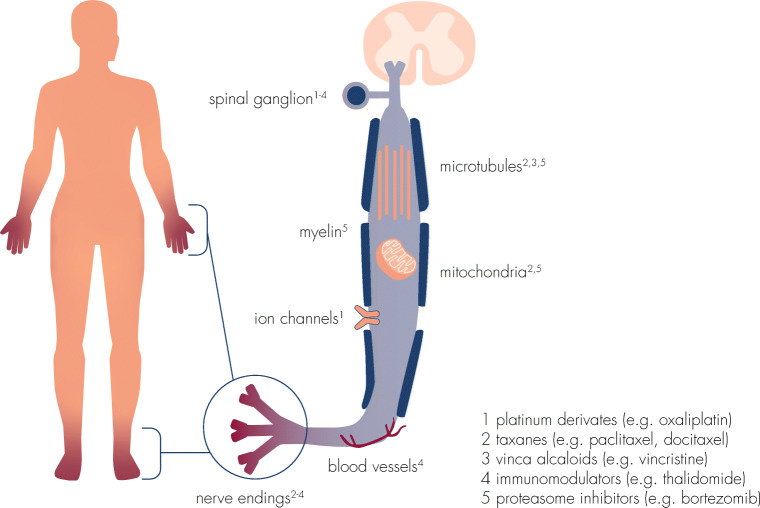
Putative targets of toxicity of chemotherapeutic agents. Neurotoxicity of different chemotherapeutic agents is mediated by interference with a variety of cellular structures and components of the peripheral nervous system, modified from [16, 17]

The pathophysiology of CIPN upon administration of anticancer therapies has been analyzed in detail by a variety of studies and reviews [11, 12, 15, 16]. Although the exact pathogenesis is still not fully understood, the underlying mechanism of CIPN is regarded to be multifactorial with various sites of involvement. Chemotherapeutic drugs exert neurotoxic effects on myelin sheets (myelinopathy), on sensory cell bodies in the dorsal root ganglion (neuronopathy), and on axonal components (axonopathy), including ion channels, microtubules, mitochondria, and associated capillaries (Fig. 1). Subsequently, common degenerative pathways are triggered leading to the production of pro-inflammatory cytokines, activation of apoptotic signaling cascades, and alteration of neuronal excitability which might consequently result in epidermal fiber loss [18, 19]. In addition to peripheral neuronal dysfunction, long-term changes in the central nervous system may result in chronic pain [15].

## Clinical presentation and diagnostics

### Prevalence and risk factors

In general, prevalence and incidence of CIPN vary according to the chemotherapeutic agent, dose, duration of exposure, and method of assessment [17]. According to metadata (31 studies with a total of 4179 patients), CIPN prevalence amounts up to 68% within the first month after chemotherapy, 60% after 3 months, and 30% after 6 months [20]. Importantly, CIPN-related symptoms often persist > 6 months after cessation of chemotherapy, and there is evidence for a relationship between QoL and the severity of neuropathy [14, 21]. Further data evaluating the long-term consequences of docetaxel treatment in breast cancer and oxaliplatin treatment in colorectal cancer patients revealed that 42% and 84% of patients, respectively, displayed CIPN symptoms 2 years after therapy [22, 23]. In addition, a systemic review found persistent CIPN-related symptoms in 11% to more than 80% of the studied breast cancer patients at 1 to 3 years after chemotherapy [14]. Thus, although overall severity of peripheral neuropathy declines and sensory nerve conduction improves over time, recovery is often incomplete.

Besides specific risk factors such as diabetes, hypothyroidism, renal insufficiency, alcohol abuse, and preexisting neuropathy (e.g., diabetic neuropathy), there are tumor-associated and therapy-related factors that contribute to the individual risk for CIPN [6]. However, nature and severity of CIPN mainly correlate with the administered neurotoxic agent, the administered cumulative dose, and the duration of exposure (Table 1).

**Table 1:** Specific toxicity profiles of neurotoxic chemotherapeutic agents, modified from [6, 17]

Cancer therapy		Increased risk of neurotoxicity	Incidence		Symptoms
Substance	Class		Grade 1–2*	Grade 3–4*	
Cisplatin^1,2^	Platinum	> 300–350 mg/m^2^	14–63%	7–21%	• Predominantly sensory neuropathy • Painful paresthesia, numbness, tingling, impaired vibration sense, sensory ataxia
Oxaliplatin^1,2,4,6^	Platinum	> 550 mg/m^2^	18–100%	12–39%	• Acute sensory symptoms and chronic sensory neuropathy • Acute cold-induced paresthesia, cramps, fasciculations
Paclitaxel^1,2,4^	Taxane	> 250–300 mg/m^2^	20–50%	6–20%	• Predominantly sensory neuropathy • Painful paresthesia, numbness • Decreased vibration or proprioception • At higher doses, myalgia and myopathy
Docetaxel^1,2,4^		> 100 mg/m^2^			
Vincristine^1,2^	Vinca alkaloid	> 2–6 mg/m^2^	35–45%		• Sensory neuropathy • Hypoesthesia (up to 100%), tingling paresthesia • Muscle cramps and mild distal weakness • Autonomic neuropathy
Thalidomide^1,5^	Immunomodulatory/antiangiogenic agent	> 20 g	≤ 83%	≤ 35%	• Sensory neuropathy • Muscle cramps and mild distal weakness
Bortezomib^1,2,3^	Proteasome inhibitor	> 16–26 mg/m^2^	≤ 50%	≤ 30%	• Painful, small-fiber sensory neuropathy • Painful paresthesia, burning sensation, sensory ataxia • Autonomic neuropathy including orthostatic hypotension
Eribulin	Microtubule inhibitor	n.a.	n.a.	n.a.	• Sensory neuropathy • Myalgia • Note: almost all patients are pretreated with (multiple) neurotoxic cancer therapies

^1^Increased single doses are associated with greater neurotoxicity

^2^Increased cumulative doses are associated with greater neurotoxicity

^3^Dose threshold relationship, increasing risk until a plateau at 40 to 45 mg/m^2^

^4^Longer infusion duration may reduce neurotoxicity

^5^Longer duration of treatment increases the risk of neurotoxicity

^6^“Stop-and-go” regimens may be associated with lower neurotoxicity

*NCI-CTCAE scale

### Evaluation of CIPN-specific symptoms and disease-related burden

**Fig. 2:**
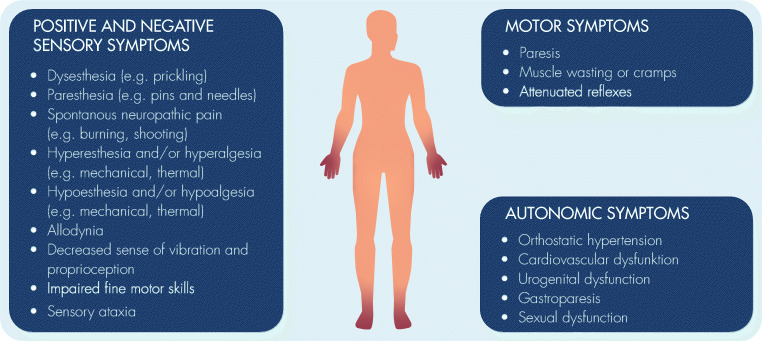
Clinical features of CIPN depending on the type of affected peripheral nerves (sensory, motor, or autonomic). CIPN is predominantly a distal symmetrical sensory neuropathy with sensory abnormalities in lower arms and lower legs (stocking/glove distribution). Motor symptoms with a similar peripheral distribution to sensory alterations are less common und usually milder. Autonomic impairment may occur but is considered to be rare

CIPN is predominantly a sensory neuropathy that may be accompanied by motor and autonomic deficits, depending on the chemotherapy regimen (Table 1, Fig. 2). Sensory dysfunctions can generate positive symptoms as well as negative symptoms.

Positive sensory symptoms can be stimulus-evoked or spontaneous and include:

- hyperalgesia (increased response to a normally painful stimulus)
- *allodynia* (increased response to a normally non-painful stimulus), resulting from thermal and mechanical (dynamic, static, and vibration) stimuli
- spontaneous (i.e., stimulus-independent, ongoing, or paroxysmal) shooting pain, electric shocks, or burning pain
- *dysesthesia* (abnormal, unpleasant, and/or painful sensation felt in the skin)
- *paresthesia* (abnormal sensation that is distracting but not generally painful)

Negative sensory symptoms include:

- reduced responses to either normally non-painful or painful stimuli in the damaged nerve territory (i.e., *hypoesthesia* or *hypoalgesia)* causing a feeling of numbness
- impaired fine motor skills (e.g., difficulties in closing buttons or holding a pen)
- disturbance of vibratory and proprioceptive sensations

The longest peripheral axons are more vulnerable to toxic drugs, and sensory symptoms usually begin in the lower limbs followed by the upper extremities, spreading from toes and fingertips in a distal symmetric sock- and glove-like distribution pattern. Most patients (approx. 68%) are simultaneously affected at upper and lower limbs, although presence of symptoms exclusively in the feet is also possible [24]. Additionally, malfunctions of the motor nervous system, e.g., paresis, cramps of the muscles, or attenuated reflexes, can occur. Motor neuropathy is more frequently seen with paclitaxel and vincristine and may manifest as weakness in arms and legs with difficulties in opening a bottle, walking stairs, or standing. In some cases, most commonly with exposure to vinca alkaloids, the autonomic nervous system is included, with symptoms like orthostatic hypotension, cardiovascular or urogenital dysfunction, and gastroparesis [17]. Since most patients receive multiple potentially neurotoxic agents, additive or synergistic effects are possible.

CIPN-associated QoL deteriorations including increased risk of falling are burdensome for patients and often underestimated by physicians [4, 25, 26]. Similarly, reports by patients and clinicians lead to highly different CIPN severity scores, and patient-reported outcomes typically reveal greater neurotoxicity than clinician assessment [27, 28]. Consequently, there is a need for assessment of both patients’ and clinicians’ perspectives to adequately rate and treat CIPN symptoms [28]. In addition, comparing CIPN symptom assessment via patient-reported outcomes and quantitative sensory testing (QST), which measures the detection threshold of sensory stimuli to quantify changes in nociceptive, thermal, and vibration perception, displayed weak-to-moderate correlations in breast cancer patients. However, with respect to pain, numbness, tingling, and reduction in tactile and vibration perception, QST outcomes appear to be more closely associated with CIPN symptoms, and thus might be considered for monitoring and support of CIPN therapy [29].

### Diagnostics

Guidelines by national and international societies recommend neurological examination of cancer patients prior to initiation of potentially neurotoxic cancer therapies in order to obtain a neurological baseline status and identify patients at risk of CIPN [6, 7]. Important clinical conditions that may pre-dispose patients for CIPN include preexisting peripheral nervous system dysfunctions such as radiculopathy, diabetic neuropathy, or Charcot-Marie-Tooth disease [16].

Once CIPN is presumed, further diagnosis is made by history and clinical presentation [5, 6]. Bedside testing for assessment of negative and positive sensory symptoms can be useful to find possible abnormalities suggestive of a relevant lesion or disease which affects the somatosensory system [30]. Decreased or increased tactile senses are best assessed with a cotton swab or wooden stick, thermal sense with warm and cold objects, and vibration sense with a tuning fork (supplemental table 1). Diagnosis can be supported by patients’ documentation of neurotoxic complaints and assessment of QoL, e.g., by using the European Organization for Research and Treatment of Cancer (EORTC), QoL Questionnaire-CIPN-twenty-item scale (QLQ-CIPN 20), or clinician-assessed neuropathy scores (e.g., Total Neuropathy Score, TNS). The QLQ-CIPN20 is a 20-item questionnaire evaluating sensory, motor, and autonomic symptoms and function with each item measured on an ordinal 1–4 scale (1, not at all; 4, very much) [31]. The complete version of the TNS has been used as a reliable tool for accessing the presence and severity of CIPN [32]. In addition, modified versions have been developed, known as TNS-reduced (TNSr) scale and TNS-clinical (TNSc) scale which have been validated in a multicenter setting in patients with CIPN [33]. Diagnosis may also be supported by application of specific tools for neuropathic pain screening such as painDETECT®, which contains nine sensory symptom items and was designed for detection and documentation of pain in practice and clinic [34]. Likewise, DN4 (Neuropathic Pain in 4 Questions), another useful screening tool to estimate the probability of neuropathic pain, contains questions to the patient as well as items based on clinical examination [35]. In addition, nerve conduction studies (NCS), electromyography (EMG), and QST can support clinical assessment of CIPN [5]. Although not routine, skin biopsies might be carried out to support diagnosis, especially in patients with neuropathic pain in whom other diagnostic tools are normal or negative, e.g., in CIPN resulting from small nerve fiber dysfunction. The method can be included in the diagnostic assessment as well as for follow-up of patients with peripheral neuropathy [36].

As some chemotherapeutic substances can provoke polyneuropathy-like symptoms, differential diagnoses, e.g., palmoplantar erythrodysesthesia (also called as “hand-foot syndrome”) after treatment with capecitabine or pegylated doxorubicin or paraneoplastic neuropathy mediated by onconeural antibodies targeting peripheral nervous system epitopes, should be considered to ensure appropriate treatment [6, 17]. Furthermore, other rare neuropathies like erythromelalgia may also cause symptoms similar to CIPN [37].

## Therapy of CIPN

### Prophylactic interventions to prevent CIPN-associated functional loss

Several compounds have been investigated in clinical studies for their efficiency in prevention of CIPN, including anticonvulsants, antidepressants, vitamins, minerals, and other chemoprotectants without reliable or conclusive clinically meaningful benefits [6, 7, 38]. Hence, there is no strong evidence for an established or recommended medical prophylaxis of CIPN. However, dose adjustment algorithms may contribute to the reduction of higher grade CIPN while maintaining treatment efficacy in individual patients [6]. Furthermore, literature data suggest extremity cooling during administration of certain chemotherapeutic agents (e.g., taxanes) as an option for reduction of severity, when CIPN development or symptom worsening can be expected [39, 40].

Upon initiation of potential neurotoxic anticancer therapies, regular functional exercises (mobility, sensorimotor, and vibration training) should be considered [6], which is in line with a growing body of literature, suggesting that physical exercise can reduce CIPN-induced symptoms and functional impairment [41–43].

### Therapy of CIPN-related neuropathic symptoms

National and international guidelines provide recommendations for non-pharmacological as well as pharmacological treatment options of manifest CIPN. The pharmacological recommendations are summarized in Table 2 and specified for therapeutic procedures as well as advising expert societies. Overall, recommendations in current guidelines are mainly based on results from studies in patients suffering from other types of neuropathic pain such as postherpetic neuralgia, painful diabetic polyneuropathy, and post-surgical neuropathic pain or on studies specific for CIPN treatment but with low-quality medical evidence.

**Table 2:** Overview of therapy recommendations for treatment of CIPN by leading guidelines of expert societies. Please note the approval status of respective drugs (status: October 2020)

Therapeutic procedure to treat CIPN Approval for pain treatment in EU/USA	Mode of action	Comments and frequent side effects	Recommendations of expert society			References
			S3 Guideline Supportive Therapy (DKG/ASORS, DGHO, DEGRO) 2017 [6]	ASCO Practice Guideline 2014 [7]	German Society for Neurology (DGN) 2019 [44]	
SSNRI						
*Duloxetine* EU: approved for painful diabetic peripheral neuropathy USA: approved for diabetic peripheral neuropathic pain	• Analgesic properties due to presynaptic reuptake inhibition of serotonin and noradrenaline • Increased activation of inhibitory system of descending nerves (pain inhibition)	• Nausea, dry mouth, somnolence, headache, anxiety	**+**	**+**	**+**	Smith et al. [45]; Binder and Baron Dtsch Arztebl Int, 2016. 113(37):616-625 Schlereth et al. [44]
*Venlafaxine* EU/USA: off-label	• Analgesic properties due to presynaptic reuptake inhibition of serotonin and noradrenaline • Increased activation of inhibitory system of descending nerves (pain inhibition)	• Nausea, dry mouth, somnolence, headache, anxiety, hypertension		n.s.	(+)	Durand et al. [46] Binder and Baron Dtsch Arztebl Int, 2016. 113(37):616-625 Schlereth et al. [44]
Tricyclic antidepressants						
*Amitriptyline*, *nortriptyline* EU: Amitriptyline: approved for neuropathic pain, nortriptyline: off-label USA: off-label	• Blocking of voltage-dependent sodium channels • Presynaptic reuptake inhibition of biogenic amines (e.g., noradrenaline, serotonin)	• Drowsiness, fatigue, dizziness, hypotension, weight gain	(+)	**-**	**+**	Kautio et al. [47] Hammack et al. [48] Binder and Baron Dtsch Arztebl Int, 2016. 113(37):616-625 Schlereth et al. [44]
Anticonvulsants						
Calcium channel modulator: *Gabapentin*—EU: approved for peripheral neuropathic pain, USA: approved for postherpetic neuralgia *Pregabalin*—EU: approved for peripheral and central neuropathic pain, USA: approved for diabetic peripheral neuropathic pain; postherpetic neuralgia; neuropathic pain associated with spinal cord injury	• Bind to voltage-gated calcium channels on nociceptive neurons in PNS and CNS with high affinity • Reduce activating calcium influx on peripheral/central neurons	• *Gabapentin*: somnolence, dizziness • *Pregabalin*: drowsiness, somnolence, peripheral edema, weight gain • Administration of gabapentin is frequently associated with vertigo and should be considered with care in case of certain functional impairments (e.g., gait disorder).	(+)	**-**	**+**	Mishra et al. [49] Rao et al. [50] Binder and Baron Dtsch Arztebl Int, 2016. 113(37):616-625
Sodium channel modulator: *Carbamazepine* EU: approved for trigeminal neuralgia and diabetic peripheral neuropathic pain, USA: approved for trigeminal neuralgia	• Stabilizes membranes at voltage-gated sodium channels on sensitized nociceptive neurons in PNS and CNS • Reduces spontaneous activity of these neurons	• Unfavorable side effect profile • Particularly, hyponatremia as well as drug interactions should be considered	**-**	**-**	**-**	Mishra et al. [49] Rao et al. [50] Binder and Baron Dtsch Arztebl Int, 2016. 113(37):616-625
Opioids and cannabinoids						
*Opioids* (e.g., *tramadol*, *oxycodone*, *tapentadol*) EU/USA: approved for moderate-to-severe pain	• Agonist effects at μ-opioid receptor in the CNS • Dependent on intrinsic activity at the receptor: segregation into low-potent (weak) and high-potent (strong) opioids • Some also act via noradrenergic and serotonergic reuptake inhibition on the inhibitory system of descending nerves (pain inhibition)	• Sedation, dizziness, headache, constipation, nausea, itch • Dependency, abuse	(+)	n.s.	(+)	Finnerup et al. [51] Nagashima et al. [52] Sommer et al. Eur J Pain, 2020. 24(1):3-18 Binder and Baron Dtsch Arztebl Int, 2016. 113(37):616-625 Schlereth et al. [44]
*Cannabinoids* EU/USA: off-label	• Agonists at CB1 receptors in CNS, spinal cord, and peripheral nerves • Act via inhibition of neuronal excitability	• Some cannabinoid compounds are psychoactive • Synthetic cannabinoid receptor agonists may have higher psychosis-inducing potential than natural cannabis and should be considered with care	n.s.	n.s.	**-**	van Amsterdam et al. J Psychopharmacol, 2015. 29(3):254-63 Schlereth et al. [44]
Topical therapies						
*Lidocaine patch (700 mg)* EU/USA: approved for postherpetic neuralgia	• Inhibits ectopic action potentials via blocking of abnormally functioning (sensitized) Nav1.7 and Nav1.8 sodium channels in the dermal nociceptors • May have anti-inflammatory properties via regulation of T cell activity and suppression of nitric oxide production • Act as a mechanical barrier to the area of allodynia, preventing stimulation	• Burning, erythema, pruritus, or skin irritations at application site • Unlike conventional lidocaine patches, lidocaine patches developed for pain relief do not cause cutaneous hypoesthesia	(+)	n.s.	(+)	Binder and Baron Dtsch Arztebl Int, 2016. 113(37):616-625
*Capsaicin patch, 179 mg (EU)/8% (US)* EU: approved for topical treatment of peripheral neuropathic pain as monotherapy or in combination with other pharmaceutical products for the treatment of pain USA: approved for postherpetic neuralgia and diabetic peripheral neuropathic pain	• Highly selective agonist of TRPV1 that induces activation of TRPV1-expressing cutaneous nociceptors • Initial TRPV1 activation results in transient ion influx (Na^+^, Ca^+^) with subsequent nerve depolarization and propagation of action potentials • Prolonged capsaicin exposure induces reversible defunctionalization of nociceptor function, thereby providing pain relief for an extended period	• Pain or erythema as well as burning sensation at application site • Adverse reactions are usually transient, self-limiting, and mild-to-moderate in intensity	(+)	(+)#	**+** (In case of localized neuropathic pain, the capsaicin patch should be considered for first-line therapy)	Filipczak-Bryniarska et al. [53] Maihöfner and Heskamp [54] van Nooten et al. [55] Binder and Baron Dtsch Arztebl Int, 2016. 113(37):616-625 Vinik et al. BMC Neurol, 2016. 16(1):251 Anand and Bley [56]
*Gel formulation (baclofen, amitriptyline and ketamine or amitriptyline and ketamine)*	• Combined mode of action, i.e., GABAergic modulation, blockade of sodium channels and glutamatergic (NMDA) receptors	Not specified	**-**	**-**	n.s.	Barton et al. [57] Gewandter et al. [58]
*Cream formulation* (1% menthol)	• Selectively activates TRPM8, which is also activated upon sensation of cold temperature and after sensory nerve injury	Not specified	(+)	**-**	n.s.	Hershman et al. [7] Fallon et al. [59]

**+**, recommended for treatment of CIPN by indicated guideline; **-**, not recommended for treatment of CIPN by indicated guideline; brackets indicate weak recommendation, e.g., due to low-quality medical evidence for CIPN and/or unfavorable side effect profile; *n.s.*, not specified; *SSNRI*, selective serotonin-noradrenalin reuptake inhibitor; *CNS*, central nervous system; *GABA*, gamma-aminobutyric acid; *NMDA*, glutamatergic N-methyl-d-aspartate; *PNS*, peripheral nervous system; *TRPV1*, transient receptor potential vanilloid 1; *TRPM8*, transient receptor potential melastatin 8

^#^CIPN-data were not yet available at publication date

Conventional systemic treatment options for neuropathic pain include antiepileptic agents, antidepressants, including selective serotonin and norepinephrine reuptake inhibitors (SNRIs), and tricyclic antidepressants, as well as opioids. However, only the SNRI duloxetine is recommended for treatment of CIPN in existing guidelines [6, 7, 44], based on modest medical evidence for efficacy in one randomized, double-blind, placebo-controlled CIPN-specific trial [45]. For all other systemic treatment options, clinical evidence in CIPN is still inconclusive (Table 2).

Systemic drugs need to be slowly titrated from a low starting dose to a dose providing best efficacy and limited side effects. Of special note in this context is the interaction potential of systemically acting substances in case of co-administration with chemotherapeutic and other therapies.

Topical treatment options for neuropathic pain and CIPN include the lidocaine patch (700 mg), the capsaicin patch (179 mg), and various gel formulations.

#### Non-pharmacological intervention

The German S3 guideline for supportive therapy in oncology patients highly recommends functional exercises (balance, sensorimotor, and fine motor skill training) [6].

#### Pharmacological intervention

##### Selective serotonin-noradrenalin reuptake inhibitor

I. Duloxetine

In patients with peripheral neuropathy of other origin than CIPN, duloxetine is administered due to established efficacy. In addition, a phase III, randomized double-blind, placebo-controlled crossover study (*n* = 231) found that duloxetine treatment resulted in greater reduction of CIPN-associated pain, particularly when induced by platinum derivatives [45]. Based on these results, duloxetine is recommended for CIPN therapy [6, 7]. However, potential drug interactions, particularly regarding the hepatic metabolism of duloxetine, should be considered for each individual patient.

II. Venlafaxine

Venlafaxine may be considered for CIPN therapy in individual cases [6, 44]. The recommendation is based on data from a small study with a limited number of patients (*n* = 24) indicating significant benefit vs. placebo, albeit occurrence of several side effects [44, 46]. In addition, based on a recent comparative study, the administration of duloxetine seems to be more effective than venlafaxine in decreasing the symptoms of CIPN [60].

##### Tricyclic antidepressants

I. Amitriptyline/nortriptyline

Two randomized placebo-controlled studies of amitriptyline (*n* = 114 and *n* = 44, respectively) revealed negative results or only discrete improvements of symptoms in CIPN patients [47, 61]. In addition, results of a phase III study regarding the use of nortriptyline in patients with cis-platinum-induced peripheral neuropathy (*n* = 51) revealed only modest and questionable benefit [48]. However, based on the limited options that are available for CIPN therapy and the demonstrated efficacy of these drugs in other neuropathic pain conditions, it is reasonable to try a tricyclic antidepressant [7]. Nevertheless, potential side effects, drug interactions, and cardiac toxicity should be considered upon risk-benefit analysis [44].

##### Anticonvulsants

I. Gabapentin/pregabalin

Although both gabapentin and pregabalin have been shown to be effective in the treatment of polyneuropathies, only limited scientific evidence for CIPN treatment exists. A phase III, randomized, double-blind, placebo-controlled crossover study of gabapentin in patients with CIPN (*n* = 115) revealed no improvement in either pain intensity or sensory neuropathy [50]. Pregabalin was shown to be superior to amitriptyline und gabapentin (as well as placebo) in a randomized cancer study with respect to neuropathic pain and side effects [49]. Common adverse reactions to pregabalin and gabapentin include dizziness, drowsiness, and somnolence; pregabalin is also more frequently associated with weight gain [51].

Similar to tricyclic antidepressant, gabapentin and pregabalin may be considered for CIPN treatment based on the limited therapeutic options and the demonstrated efficacy of these agents in other neuropathic pain conditions [6].

II. Carbamazepine

Carbamazepine cannot be recommended for treatment of neuropathic pain in general due to low evidence and frequent side effects. However, administration can be considered in single cases and is generally recommended in trigeminal neuralgia [44].

##### Opioids

Overall, the response of neuropathic pain to opioid therapy is low [51]. Administration of oxycodone during chemotherapy was associated with a lowered incidence of CIPN [52]. According to the German Society for Neurology, low- as well as high-potency opioids might be considered for treating neuropathic pain of any origin as third-line option, although side effects, development of tolerance, and misuse might be limiting [44].

##### Non-opioid analgesics

Non-opioid analgesics such as non-steroidal anti-inflammatory drugs (NSAIDs), metamizole, or paracetamol display low efficacy in the treatment of neuropathic pain and are considered to be associated with a variety of potential side effects [6, 44]. However, peripheral nerve damage and neuropathic pain may arise from tissue pressure increase due to swelling of the feet and/or hands and has been described as a result of chronic venous insufficiency [62]. In these cases, NSAID therapy may be effective in reducing swelling and corresponding pain.

##### Cannabinoids

Clinical trials provide little medical evidence that cannabinoid-based medicines are effective for the treatment of neuropathic pain [44, 63]. Data from a randomized, double-blind, placebo-controlled pilot study in 16 patients could also not support a substantial benefit of cannabinoids in treating CIPN [64]. According to the guideline of the German Society for Neurology, cannabinoids are not recommended for treatment of neuropathic pain of any origin as efficacy is low and the rate of side effects is high. In individual cases and upon failure of other therapeutic options, treatment with cannabinoids may be considered in the context of a multimodal pain therapy [44].

#### Pharmacological intervention—topical therapies

##### Patches

I. Lidocaine

The lidocaine patch (700 mg) is approved for the treatment of postherpetic neuralgia but have also been suggested by guidelines for the treatment of localized neuropathic pain of other origins, including CIPN [6, 44, 65]. However, randomized clinical trials proving efficacy in CIPN are missing to date [6]. Lidocaine patches might be used as second-line option for treatment of peripheral neuropathic pain, especially in case of intolerability of oral medications (e.g., in elderly patients) [44].

II. Capsaicin

In Europe, the capsaicin patch (179 mg) is approved for topical treatment of peripheral neuropathic pain as monotherapy or in combination with other pharmaceutical products for pain treatment [66]. The guideline of the German Society for Neurology recommends the capsaicin 179 mg patch for any kind of neuropathic pain as a second-line therapy. The effect is comparable to established oral medications with good tolerability and beneficial safety profile and low risk of systemic side effects [44, 55, 67]. Thus, for localized neuropathic pain, the guideline considers primary use [44]. Subgroup-specific data from 15 patients within the QUEPP study [54] support efficacy of the capsaicin patch specifically in CIPN (s. data below), which results in recommendation of the patch in respective guidelines (Table 2) [6]. Recently, the high-dose capsaicin patch was efficiently used for pain reduction in CIPN patients in two open-label single-center studies (*n* = 18 and *n* = 16, respectively) [53, 68]. However, there is no recommendation for capsaicin treatment with low-dose formulations (< 1%) or in case of non-painful polyneuropathies [6].

##### Gel and cream formulation

I. Baclofen (0.8%), amitriptyline (3%) and ketamine (1.5%)/amitriptyline (4%), and ketamine (2%)

Topical therapy of CIPN with a gel formulation containing baclofen (0.8%), amitriptyline (3%), and ketamine (1.5%) may be suggested as it was beneficial in comparison to placebo in a randomized, double-blind, placebo-controlled trial in 208 CIPN patients. However, the overall effect was modest and not clinically relevant [57]. Furthermore, the gel is not available, and type and composition are unknown [6]. A clinical study with topical amitriptyline (4%) and ketamine (2%) twice daily for 6 days in 462 cancer survivors with CIPN revealed no significant effect on pain, numbness, or tingling [58].

II. Menthol (1%)

Significant reduction of pain as well as some improvement in functionality and sensitivity was detected upon topical therapy of CIPN with 1% menthol in a proof-of-concept study with 51 patients. However, the patient cohort was small, and the study was non-blinded [59].

## Current evidence for topical treatment of CIPN with capsaicin 179 mg patch

Capsaicin is a highly selective agonist of the transient receptor potential vanilloid 1 (TRPV1) ion channel and induces activation of TRPV1-expressing cutaneous nociceptors. Upon activation, TRPV1 opens transiently resulting in an ion influx (Na^+^, Ca2^+^) with subsequent release of vasoactive neuropeptides, depolarization, and propagation of action potentials into spinal cord and brain. Patients may experience this as various sensations at the application site (warming, burning, stinging, or itching) [56]. Importantly, while environmental or inflammatory stimuli result in transient TRPV1 activation, prolonged exposure of the chemically stable capsaicin induces a cascade of cellular events (e.g., cytoskeleton breakdown and loss of mitochondrial function) that result in reversible nociceptor defunctionalization within the (painful) application area, providing control of localized neuropathic pain symptoms for an average of 5 months [56, 69]. Treatment with the capsaicin patch may be repeated every 3 months or later as needed [66]. Re-treatment after 2 months is possible for individual patients after careful assessment by the physician. Interestingly, with multiple applications, pain reduction can steadily increase with each application and re-treatment times can get longer [70, 71].

Exposure to the capsaicin 179 mg patch reduced the density of epidermal nerve fibers (ENF) and induced modest but significant changes in tactile thresholds as well as sharp mechanical pain detection in healthy volunteers. Tactile and sharp pain sensations returned to normal within 12 weeks after application, and ENF density was regenerated 24 weeks after exposure. The latter suggests that the proximal dermal origins of ENFs remained intact and that only the distal dermal nerve segments and epidermal nerve endings are affected [72].

**Fig. 3:**
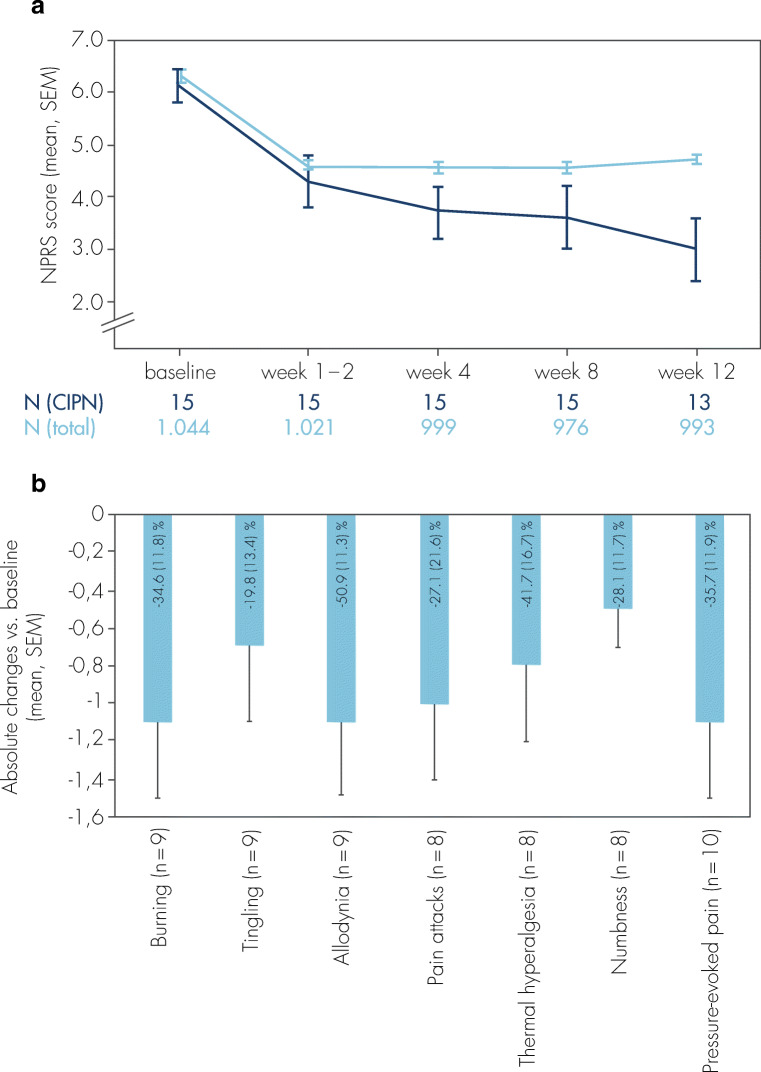
**a** Average pain intensity (NPRS 0–10 scores) before and within 12 weeks after single treatment with the capsaicin 179 mg patch in patients with CIPN-related pain (dark blue) and in the total study population (light blue; peripheral neuropathic pain of different etiologies). The absolute change between the mean NPRS scores at baseline and week 1–2 to week 12 was − 2.4 (0.4 SEM, *p* ≤ 0.001, paired *t* test) for CIPN patients and − 1.7 (0.1 SEM, *p* ≤ 0.001, paired *t* test) for the total QUEPP study population. NPRS = numeric pain rating scale; SEM = standard error of the mean. **b** Mean scores of intensity of sensory symptoms between baseline and the end of the observational period at week 12 in CIPN patients assessed with the painDETECT® questionnaire. For each symptom, single scores were assessed (never, 0; hardly noticed, 1; slightly, 2; moderately, 3; strongly, 4; very strongly, 5) at baseline and after 12 weeks. Relative changes of symptoms (%) are shown within the bars (mean, SEM). Baseline score > 0; SEM = standard error of the mean. Modified from [73]

Data from a CIPN subgroup (*n* = 15) within the open-label, clinical observational QUEPP study [54] point to similar efficacy results in comparison to the total study population (*n* = 1044 patients with various peripheral neuropathic pain conditions). The average pain intensity assessed by the 11-point numeric pain rating scale (NPRS) continuously decreased over the course of the study (Fig. 3a); a reduction of pain intensity for ≥ 30% was achieved in 46.7% of the CIPN patients, and pain reduction ≥ 50% was reported for 33.3% of the CIPN patients [73, 74]. Tolerability was accessed as either “very good” or “good” by 80% of the treating physicians (supplemental figure 1). In addition, therapy with the capsaicin patch also improved symptoms typically associated with neuropathic pain, including tingling and burning sensation, thermal hyperesthesia (especially cold sensitivity), and numbness (Fig. 3b). Thus, although the evidence is low due to the small number of participants, the results for CIPN patients are similar to those of the overall QUEPP study population.

Analyses of the total QUEPP population (*n* = 1.044) revealed that the analgesic effect of the capsaicin patch is higher and lasts longer in patients with shorter durations of preexisting neuropathic pain. The highest response to capsaicin treatment was achieved within 6 months of onset of peripheral neuropathic pain. Therefore, treatment should be initialized as early as possible [75].

Data from a small single-center study with 18 patients suffering from oxaliplatin-induced neuropathy also confirmed efficacy of the capsaicin 179-mg patch in pain reduction [53]. Interestingly, recent data reveal even further properties of capsaicin beyond significant reduction of CIPN-related neuropathic pain: The study published by Anand et al. suggests a disease-modifying property of capsaicin therapy via regeneration and phenotypical restoration of sensory nerve fibers in CIPN patients in remission after chemotherapy [68]. This was demonstrated by skin biopsy and detailed immunohistochemical analyses before and after capsaicin treatment. It has been suggested that the capsaicin-induced defunctionalization of abnormal nerve fibers may support subsequent nerve regeneration.

## Treatment algorithm for clinical practice

**Fig. 4:**
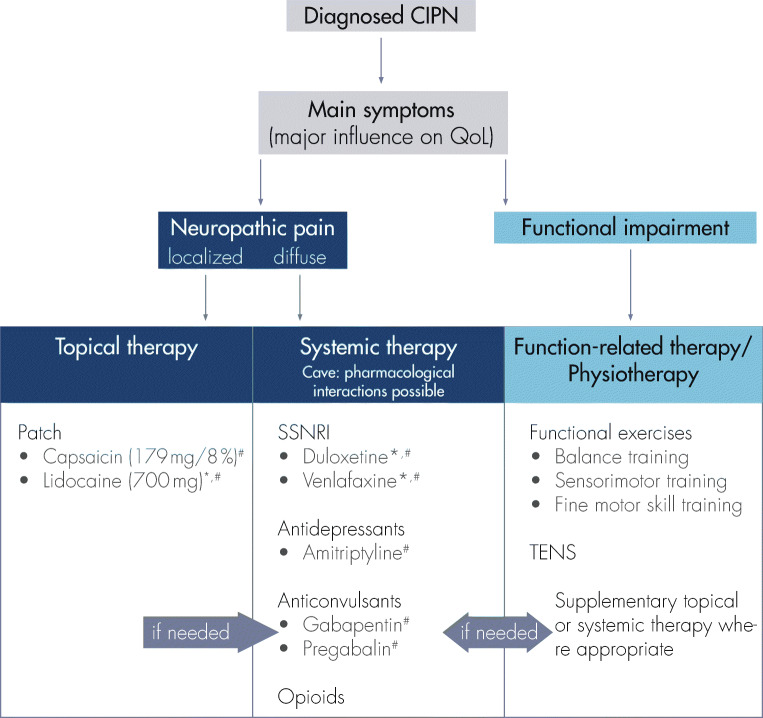
Treatment criteria for clinical practice upon CIPN diagnosis. Please note off-label use for treatment of CIPN in EU (*)/USA (^#^) (for details, see Table 2)

Treatment of diagnosed CIPN should address the symptoms with the highest impact on patients’ QoL. It appears reasonable to distinguish between neuropathic pain and functional impairment without pronounced pain symptoms. A treatment algorithm for CIPN therapy in clinical practice is proposed in Fig. 4. If pain is the major reason for deteriorated QoL, pharmaceutical treatment options are appropriate, while functional impairment without severe or limiting pain is reasonably treated with physiotherapeutic methods. However, since individual patients might simultaneously be affected by pain and functional impairment, combination therapies are feasible. In addition, since CIPN-mediated symptoms are typically located in hands and/or feet, it is reasonable to consider a local treatment option in the first place [44].

Since systemic therapies might be accompanied by systemic interactions and side effects, the choice of therapy should be influenced by comorbidities and co-medications. Of special note in this context is the administration of pregabalin or gabapentin in patients with sensory ataxia (especially in case of non-significant pain symptoms) as these drugs might induce dizziness and subsequently increase the risk of falling.

Consequently, and in view of patients with comorbidities or at risk of cancer recurrence, topical treatment options with a non-systemic mode of action should be preferred for patients with CIPN-associated pain due to the low rate of systemic side effects and systemic drug interactions.

## Conclusion

CIPN represents a major concern in cancer therapy. There is limited evidence-based data available for prophylaxis and treatment of this condition. Early start of functional exercises during chemotherapy is recommended.

Existing data on pharmaceutical treatment of CIPN allow weak recommendations for duloxetine. Given the paucity of available alternative treatment strategies and extrapolating efficacy data from other neuropathic pain conditions, certain antidepressants (amitriptyline), and anticonvulsants (gabapentin, pregabalin) may be considered. If appropriate, opioids may be used on the basis of clinician’s experience.

Systemic treatments are associated with a typical tolerability profile and carry the risk of drug interactions. Study results from open-label trials provide evidence for successful topical treatment of CIPN with capsaicin 179 mg patch. The patch provides significant reduction of CIPN-related pain and symptoms as shown in the literature for other neuropathic disorders, while displaying low rates of side effects [54, 67, 70]. In particular, systemic side effects and drug interactions can be reduced or avoided. In addition, recent data point to promising disease-modifying effects of the capsaicin patch [68].

## Supplementary information

ESM 1 (DOCX 186 kb)

## Data Availability

Data sharing is not applicable to this article as no datasets were generated or analyzed during the current study.
