# Supplementary material for: Chemotherapy-induced peripheral neuropathy (CIPN): current therapies and topical treatment option with high-concentration capsaicin
Source: Support Care Cancer. 2021 Feb 23;29(8):4223–38. doi: 10.1007/s00520-021-06042-x (PMC8236465; doi:10.1007/s00520-021-06042-x)
Supplement: Supplementary file 1 — (DOCX 186 kb) [file 520_2021_6042_MOESM1_ESM.docx]

**Supplemental table 1: Definition and assessment of typical negative and positive sensory symptoms and signs in patients with neuropathic pain.** Modified from [1].

| **Symptom** | **Definition** | **Bedside Assessment** | **Response** |
| --- | --- | --- | --- |
| **Negative symptoms** | | | |
| **Hypoesthesia** | Reduced sensation of normally non-painful stimuli | Soft brush or cotton swab | Reduced perception, numbness |
| **Hypoalgesia** | Reduced sensation of normally painful stimuli | Pinprick, wooden stick | Reduced perception, numbness |
| **Pallhypoesthesia** | Reduced sensation of a vibration stimulus | Turning fork on joint, bone or soft tissue in the region of pain | Reduced perception threshold |
| **Thermohypoesthesia** | Reduced sensation of cold or warm stimuli | Warm (~ 45°C) and cold (~ 10°C) objects (e.g. metal roller, glass with water) | Reduced perception |
| **Positive symptoms (spontaneous or evoked sensation of pain)** | | | |
| **Paresthesia** | Distracting and uncomfortable, non-painful abnormal sensation with no apparent physical cause (e.g. tingling, pins and needles) | Grade intensity (0-10); area in cm^2^ | Not applicable |
| **Dysesthesia** | Unpleasant or painful abnormal sensation, spontaneous or evoked (e.g. burning, prickling) | Grade intensity (0-10); area in cm^2^ | Not applicable |
| **Spontaneous pain** | Stimulus-independent ongoing pain (burning, squeezing, pressure) and paroxysmal pain (stabbing, electric shocks) | Grade intensity (0-10); area in cm^2^ | Not applicable |
| **Allodynia** | Increased response to normally not painful stimuli | Soft brush or cotton swab | Painful sensation |
| **Hyperalgesia** | Increased response to painful stimuli (mechanical, heat or cold) | Pinprick, wooden stick, warm and cold objects | Painful sensation |


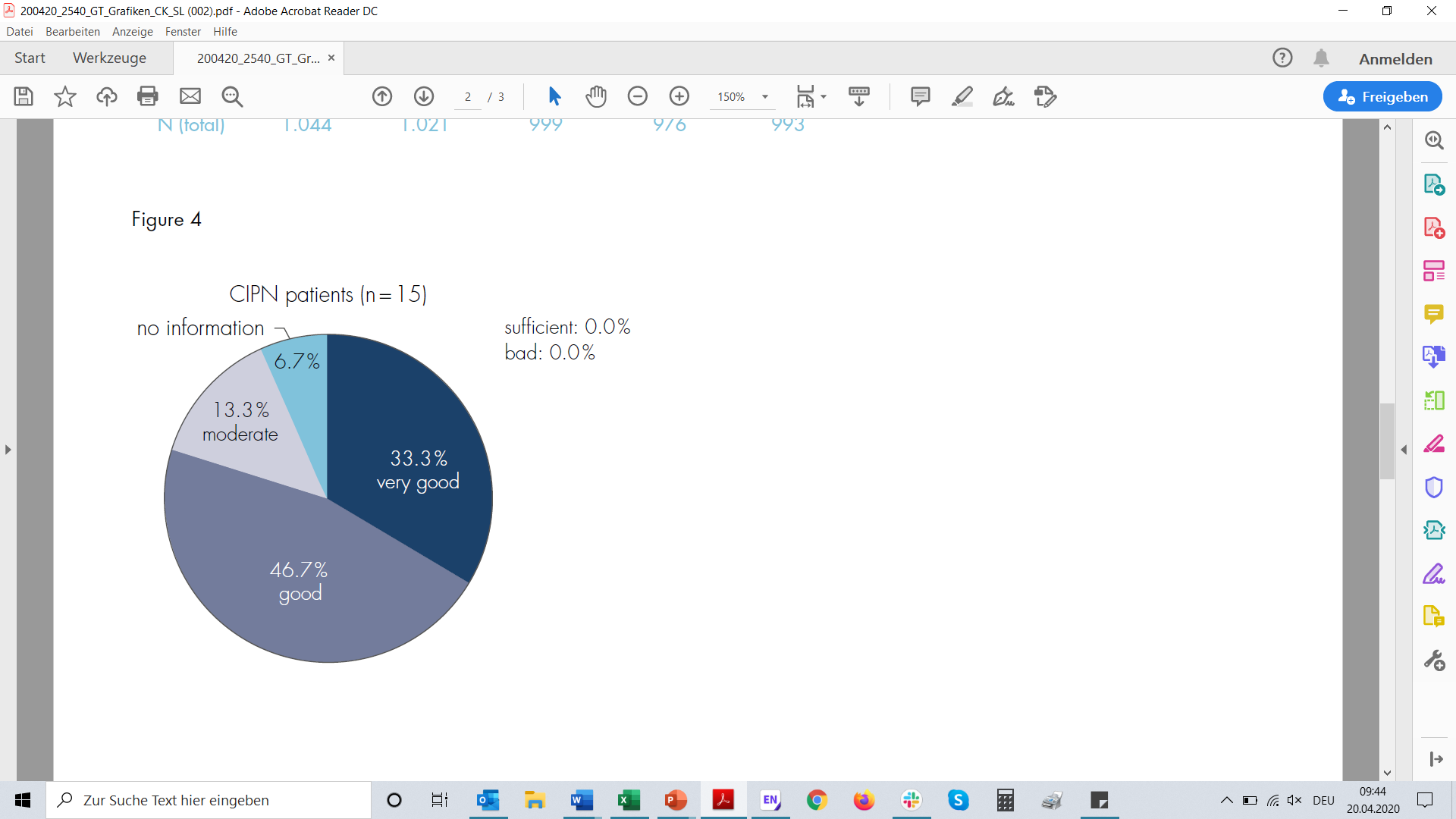


**Supplemental figure 1: Physicians' assessment of tolerability of the treatment with the capsaicin 179 mg patch in patients with CIPN (n = 15).** At the end of the observation period physicians could evaluate the tolerability of the treatment as very good, good, moderate, sufficient, or bad. Modified from [2, 3].

**Supplemental Reference:**

1. Baron, R., A. Binder, and G. Wasner, *Neuropathic pain: diagnosis, pathophysiological mechanisms, and treatment.* Lancet Neurol, 2010. **9**(8): p. 807-19.
2. Hustedt, I.W., M.L. Heskamp, and C.G. Maihöfner, *Treatment of chemotherapy-induced polyneuropathies: Effectiveness and safety of the Capsaicin 8 % cutaneous patch.* WCP (IASP) Buenos Aires 6-11 Oct 2014, poster abstract no. 3082, poster no. PF-434. 2014.
3. Hustedt, I.W., M.L. Heskamp, and C.G. Maihöfner, *[Effectiveness and safety of local treatment of painful chemotherapy-induced polyneuropathies with capsaicin 8 % cutaneous patch].* Deutscher Schmerzkongress, Hamburg, Germany, 22-25 Oct 2014, poster abstract no. 258, poster no. P05.06. 2014.
